# Cord Blood Transplantation: Can We Make it Better?

**DOI:** 10.3389/fonc.2013.00238

**Published:** 2013-09-17

**Authors:** Leland Metheny, Paolo Caimi, Marcos de Lima

**Affiliations:** ^1^Stem Cell Transplantation Program, University Hospitals Case Medical Center, Case Western Reserve University, Cleveland, OH, USA

**Keywords:** umbilical cord blood transplantation, *ex vivo* expansion, delayed engraftment, double umbilical cord transplant, mesenchymal stem cells, reduced intensity conditioning

## Abstract

Umbilical cord blood is an established source of hematopoietic stem cells for transplantation. It enjoys several advantages over bone marrow or peripheral blood, including increased tolerance for Human Leukocyte Antigen mismatches, decreased incidence of graft-versus-host disease, and easy availability. Unrelated cord blood does have limitations, however, especially in the treatment of adults. In the 24 years since the first umbilical cord blood transplant was performed, significant progress has been made, but delayed hematopoietic engraftment and increased treatment-related mortality remain obstacles to widespread use. Here we summarize the latest results of unrelated cord blood transplants, and review strategies under investigation to improve clinical outcomes.

## Introduction

Since the first sibling human leukocyte antigen (HLA)-matched umbilical cord blood transplant (UCBT) was performed in 1989 by Gluckman and Colleagues on a pediatric patient with Fanconi’s anemia, cord blood stem cells have been recognized for their benefits (1). Umbilical cord blood (UCB) hematopoietic stem cells can be procured easily, without risk to the donor, and an appropriate number of stem cells can be stored, tested, and processed for future use (2–4). These advantages make UCB suitable to fill the growing need for suitable grafts for patients with hematologic malignancies or underlying bone marrow or metabolic defects. It is also important to note that a significant population of patients, specifically minority groups, lack a suitable HLA-matched bone marrow, or peripheral stem cell donor, thus UCB offer an important source of allografts for such groups (5). In this review we examined the two decade history of this subset of allogeneic stem cell transplants, and then focused specifically on the major developments for improving and accelerating cord blood engraftment, widely recognized as its’ Achilles heel. A comparison of haploidentical and cord blood grafts is beyond the scope of this review.

## Pediatric Umbilical Cord Blood Transplantation

Following the report of the first sibling HLA-matched UCBT, the first pediatric studies showed UCBT present higher rates of primary engraftment failure as well as delayed time to engraftment. Initial studies investigating the impaired engraftment in UCBT focused on HLA matching and the optimum dosage of CD34+ cells or total nucleated cells (TNC). It is important to note that many of these studies were retrospective; almost 20 years passed between the Gluckman’s first transplant and the first prospective randomized clinical trial (phase 2). This underscores the continued need for prospective randomized clinical trials, as bias is a much more significant problem in retrospective studies.

Wagner et al. described 44 patients who underwent matched sibling UCBT, mismatched at 0 or 1 HLA locus (6). Day 50 engraftment rate was 85%, while the rate of grade II–IV acute graft-versus-host disease (aGVHD) was a very low 3%, with a survival rate at 1.6 years of 72%. However, primary graft failure occurred in 15% of patients. Subsequently, 22 patients were reported that received either HLA-matched or HLA 1–3 antigen mismatched cord grafts (7). Engraftment at day 50 was 100%, with aGVHD rate of 11%, while 6-month survival rate was 65%. These early studies positioned UCBT as a clear alternative for pediatric patients in need of allogeneic transplantation.

Subsequently, Barker and Coworkers compared 0–3 HLA mismatched UCBT recipients with recipients of unrelated matched bone marrow transplants (BMT). This matched pair analysis suggested that mismatched UCBT led to similar survival than matched BMT, but at expense of longer time to engraftment with similar aGVHD rates (8). Rubinstein et al. performed an analysis of transplants from the New York cord blood bank, and observed that the chances of successful engraftment could be increased with a better HLA-matched cord (9).

Gluckman et al. reported the Eurocord registry experience in 1997, showing that recipients of related UCBT had rates of 1-year survival, grade II-IV aGVHD, and day 60 neutrophil engraftment of 55, 18, and 79%, respectively; recipients of unrelated UCBT had rates of 1-year survival, grade II–IV aGVHD, and neutrophil engraftment at day 60 of 31, 32, and 89% (10). Rocha et al. retrospectively compared outcomes of pediatric UCBT, T cell depleted bone marrow, and non-manipulated BMT. UCB was again associated with delayed neutrophil and platelet engraftment as well as increased early treatment-related mortality (TRM). However, the risk of aGVHD was lower, while the overall risk of relapse was similar among groups. Mortality was greatest in the T cell depleted BMT group (11).

In another observational, retrospective study, Eapen and Collaborators compared pediatric matched and mismatched UCBT with matched or mismatched unrelated BMT. The authors again pointed to delayed engraftment and increased TRM with UCBT, especially for those receiving low cell doses. However, 5-year leukemia-free survival (LFS) rates were similar between all groups and the risk of relapse was lowest in UCBT with two mismatched alleles (12).

The first prospective multicenter study in pediatric UCBT, published in 2008, enrolled 191 patients with high-risk hematologic malignancies. Cord grafts were matched to at least 3 of 6 HLA loci, and median TNC infused was 3.9 × 10^7^ c/kg. The conditioning regimen was myeloablative, with 1350 cGy total body irradiation (TBI), cyclophosphamide (60 mg/m^2^), and equine ATG (15 mg/kg). Median time to neutrophil and platelet engraftment was 27 and 173 days, respectively. Primary graft failure occurred in 11% of patients, while rates of grade II–IV aGVHD, relapse, and 2-year OS were 42, 19, and 50%, respectively (13).

Two subsequent studies emphasized the importance of cell dose on engraftment and TRM. In an observational study of pediatric UCBT, Wagner’s group found that among donor-recipient pairs with ≤2/6 HLA mismatches at HLA A, B, and DRB1, UCBT consisting of a cell dose of 1.7 × 10^5^ CD34+ cells/kg resulted in improved outcomes (14). Gluckman, Locatelli, and others on behalf of the Eurocord, showed that pediatric leukemia patients receiving a TNC dose above 3.7 × 10^7^ had a higher probability of engraftment and survival (10, 15, 16), with a median time to neutrophil engraftment of 25 days compared to 35 days for those receiving <3.7 × 10^7^/kg.

These studies solidified the role of UCBT as an alternative to traditional forms of transplant. They also clearly defined the importance of HLA matching and cell dose in UCBT. Grafts with more than 3/6 HLA mismatches (low resolution typing for HLA A and B, and high-resolution DRB1 typing) result in significantly higher TRM rates and should be avoided. The importance of “minimum” cell dose was also established, and is an additional complicating factor in adults, as the cell dose threshold is unlikely to be reached with a single UCB unit in this patient population.

## Adult Umbilical Cord Blood Transplantation

Adult UCBT studies have also been mostly retrospective, and continue to show worse outcomes when compared with those of pediatric populations. The cell doses in these studies have been significantly lower than the “minimum” defined by Wagner, Gluckman and others (10, 14–16).

In 2001, Laughlin and Colleagues retrospectively analyzed the outcomes after adult UCBT following myeloablative conditioning for malignant and non-malignant hematological diseases. The median infused TNC was 1.6 × 10^7^/kg; all patients had a graft with three or less HLA mismatches. The median time to neutrophil and platelet engraftment was 27 and 99 days, respectively. Graft failure occurred in 10% of patients, and grade II–IV aGVHD rate was 60%. At 40 months, 26% of patients were alive. CD34+ cell dose was associated with improved event-free survival (EFS) (17). Subsequently, other adult UCBT studies have also observed a high rate of graft failure and delayed engraftment compared to bone marrow or peripheral blood grafts (18–22) (Tables 1 and 2).

**Table 1:** **Comparison of multiple UCBT studies, including both pediatric and adult patients and single UCBT and dUCBT**.

Study	Type	No. patients	Cord information	Primary graft failure (%)	Time to neutrophil and platelet engraftment (days)	Grade II–IV aGVHD (%)	Survival
Wagner et al. (6)	Pediatric	44	Single cord HLA identical/HLA-1 mismatch	15	Neutrophil = 22	3	1.6 year OS = 72%
Wagner et al. (7)	Pediatric	18	Single cord HLA-matched versus HLA (1–3) mismatched	0	Neutrophil = 24; platelet = 54	11	6 month OS = 65%
Wagner et al. (14)	Pediatric retrospective	102	Single cord 0–3 HLA mismatched	5	GCSF(+) neutrophil = 21; GCSF(−) neutrophil = 31; platelet (50k) = 86	11	1 year OS = 58%
Kurtzberg et al. (13)	Pediatric	191	Single cord 0–3 HLA mismatched	11	Neutrophil = 27; platelet (50k) = 174	19.5	2 year OS = 49.5%
Laughlin et al. (17)	Adult	68	Single cord 0–3 HLA mismatched	7	Neutrophil = 27; platelet = 99	60	40 month OS = 27%
Sanz et al. (18)	Adult	22	Single cord 0–3 HLA mismatched	0	Neutrophil = 22; platelet = 69	73	8 month OS = 54%
Takahashi et al. (20)	Adult	68	Single 0–2 HLA mismatched	8	Neutrophil = 22; platelet = 40	44	2 year DFS = 74%
Rocha et al. (23)	Adult retrospective	98	Single cord 0–3 HLA mismatched	20	Neutrophil = 26	26	2 year OS = 36%
Cornetta et al. (19)	Adult	34	Single cord 0–2 HLA mismatched	34	Neutrophil = 42; platelet = 180	34	D180 OS = 30%
Brunstein et al. (41)	Adult RIC	110	85% dUCBT/15% single 0–2 HLA mismatched	6	Neutrophil = 22; platelet (50k) = 59	59	3 year OS = 26%
Ballen et al. (21)	Adult RIC	21	dUCBT 0–2 HLA mismatched	9	Neutrophil = 20; platelet = 41	40	2 year OS = 71%

GCSF, granulocyte colony stimulating factor; OS, overall survival; DFS, disease free survival.

**Table 2:** **Comparison of graft sources**.

Study	Type	Groups	No. patients	Neutrophil (ANC = 500) and platelet engraftment (20,000)	Grade II–IV aGVHD	Survival
Hamza et al. (22)	Adult retrospective	UCB, at least HLA 3/6 matched	28	Neutrophil = day 29	33%	EFS day 100 = 34%; EFS 3 year = 25%
		MUD	23	Neutrophil = day 14	27%	EFS day 100 = 78%; EFS 3 year = 35%
Eapen et al. (24)	Adult retrospective/Cox regression	UCB at least HLA 4/6 matched	165	Neutrophil = day 24, platelet = day 52	30%	LFS = 59%
		PBPC	888	Neutrophil = day 14, platelet = day 19	8/8 HLA matched = 48%; 7/8 HLA matched = 52%	8/8 HLA matched = 57%; 7/8 HLA matched = 66%
		BM	472	Neutrophil = day 19, platelet = day 28	8/8 HLA matched = 39%; 7/8 HLA matched = 46%	8/8 HLA matched = 56%; 7/8 HLA matched = 57%

UCB, umbilical cord blood; MUD, matched unrelated donor; PBPC, peripheral blood progenitor cells; BM, bone marrow.

Rocha compared the outcomes of 98 UCBT patients reported to the Eurocord with those of 584 patients who received matched bone marrow grafts (intermediate resolution HLA typing for HLA A and B, and high-resolution typing for HLA-DRB1). Of note, the average TNC dose differed by a factor of 10 between groups: in the UCBT cohort the TNC was 2.3 × 10^7^ c/kg compared to 2.9 × 10^8^ c/kg for those undergoing BMT. Although the degree of donor-recipient HLA mismatches was higher in the cord blood group (most subjects received 4/6 matched units), aGVHD risk was significantly lower compared to the BMT cohort. Neutrophil engraftment was significantly delayed in the cord blood group, and primary graft failure occurred in 20% of UCBT patients versus 7% in the bone marrow group. Nevertheless, overall survival and the rates of TRM, cGVHD, relapse, and LFS were not significantly different between the two groups (23).

In a retrospective analysis using registry databases, Eapen compared 165 UCBT, 888 peripheral blood progenitor cell (PBPC), and 472 BMT recipients. TRM was higher in the umbilical cord group, but LFS was similar across all groups. The rate of aGVHD was lower after UCBT compared to PBPC, while cGVHD was lower in the UCBT compared to 8/8 matched BMT (24). A similar retrospective study by Eapen also demonstrated the importance of HLA C matching in UCBT (25) (Table 3).

**Table 3:** **Retrospective analysis of HLA C typing in UCBT**.

Study	Type	Groups	No. patients	Transplant related mortality	Overall survival
Eapen et al. (25)	Allo pediatric retrospective/Cox regression	HLA A, B, C, and DRB1 matched	69	3 year TRM = 9%	3 year OS = 57%
		HLA A, B, and DRB1 matched; HLA C mismatched	23	3 year TRM = 26%	3 year OS = 51%
		Either HLA A, B, or DRB1 mismatch + HLA C mismatch	234	3 year TRM = 31%	3 year OS = 37%

TRM, treatment-related mortality; OS, overall survival.

Taken in aggregate, these studies indicate that adults receiving single, mismatched UCB grafts have higher early mortality but similar overall survival to that observed in recipients unrelated donor BMT or PBSC. Neutrophil and platelet engraftment is delayed, and a smaller proportion of patients become platelet transfusion independent. Primary graft failure rates are also higher with single UCBT than when using peripheral blood or bone marrow grafts (Table 2). Of note, all comparative studies published to date are retrospective, and there seems to be a significant variation in center outcomes.

## Reducing TRM in UCBT: Reduced Intensity Conditioning, Double Umbilical Cord Transplants, and HLA Matching

Due to high TRM rates, UCBT is often used as a transplant of last resort. Early efforts to reduce TRM focused on reducing conditioning regimen toxicity and increasing UCB cell dose. Most studies available correspond to small prospective trials, usually from a single institution.

Reduced intensity conditioning has reduced early mortality in allogeneic transplants, and has allowed treatment of older and frailer patients (26–34). Barker et al. treated 43 patients with high-risk hematologic malignancies. Two regimens were used: busulfan (8 mg/kg), fludarabine (200 mg/m^2^), and of TBI (200 cGy) (BFT) in 21 patients; and cyclophosphamide (50 mg/kg), fludarabine (200 mg/m^2^), and TBI (200 cGy) (CFT) in 22 patients. UCB units were matched to at least 4/6 HLA loci. Median time to neutrophil engraftment was 9.5 and 22 days in the CFT and BFT groups, respectively, while sustained engraftment was observed in 94 and 76% in the CFT and BFT groups. For the whole cohort, 1-year overall survival was 39% with 21% relapse rate and 9% grade II–IV aGVHD (35). This low dose TBI-based regimen is now widely used in the U.S., and retrospective comparisons have suggested it is superior to alkylating-based combinations (36).

Double UCBT (dUCBT) has been proposed as a strategy to increase cell dose and possibly improve outcomes (37, 38). Barker and Collaborators treated 22 hematologic malignancy patients with myeloablative conditioning and dUCBT grafts containing a median TNC dose of 3.5 × 10^7^/kg, matched to at least 4/6 HLA loci. Median time to neutrophil engraftment in this adult cohort (median weight of 73 kg) was 23 days. There were no primary or secondary graft failures, while aGVHD II–IV and 1 year DFS rates were 65 and 57%, respectively (39).

MacMillan and coworkers retrospectively compared outcomes of single versus dUCBT treated at the University of Minnesota. Although cGVHD rates were similar (17 versus 18%), grade II–IV aGVHD rates were greater in the dUCBT group (58 versus 39%, *p* < 0.01). dUCBT recipients presented GVHD at earlier time points (day 28 versus 36) but had lower 1-year TRM rates (24 versus 39%, *p* = 0.02) (40).

Brunstein and Colleagues reviewed the results of a non-myeloablative conditioning regimen consisting of fludarabine (200 mg/m^2^), cyclophosphamide (50 mg/m^2^), and a single fraction of 200 cGy TBI on 110 patients with high-grade hematologic malignancies (41). TNC dose target was 3.0 × 10^7^/kg, and patients were to receive a dUCBT if a single unit did not provide that cell dose. Eight-five percent of patients received a dUCBT. TRM rate was 19% at 180 days and OS was 26% at 3 years. Relapse rates were higher in the single cord group compared to the dUCBT group (41 versus 30%, *p* = 0.07), while aGVHD incidence tended to be higher in the dUCBT cohort (62 versus 41%, *p* = 0.09). Primary and secondary graft failure occurred in 6 and 7% of patients, respectively. Overall, higher rates of TRM were associated with age below 45 years, recipient negative CMV serostatus, use of ATG, high-risk clinical features and recent fungal infection.

Kindwall-Keller conducted a prospective study involving high-risk patients with hematologic malignancies comparing single (*n* = 27) versus dUCBT (*n* = 23). Both patient groups received fludarabine (35 mg/m^2^), cyclophosphamide (1 g/m^2^), horse ATG (30 mg/kg), and 200 cGy TBI. Single UCB graft had at least 2.5 × 10^7^ TNC/kg, while dUCBT grafts should provide a combined 3.0 × 10^7^ TNC/kg. Each cord was matched at least 4/6 HLA loci to the recipient. Seven patients (26%) in the single unit group and five patients (22%) in the double unit group failed to engraft. The median time to neutrophil engraftment was 25 days in the single cord group and 23 in the double cord group (*p* = 0.99). The median time to platelet engraftment was 39 days in the single cord group and 57 in the double cord group. Relapse rates were significantly lower in the dUCBT group (30 versus 59%, *p* = 0.045). There was no significant difference in aGVHD rates between the two groups. Overall survival at 60 months was 39% in the dUCBT group and 26% in the single unit group (*p* = 0.86) (42).

Although the controversy surrounding single versus dUCBT in adult patients is far from resolved, a recently completed phase III clinical trial comparing single and double cord transplants in pediatric patients showed no difference in 1 year survival (71 versus 65%, *p* = 0.13). In addition, increased aGVHD rates were observed after dUCBT (14 versus 23%, *p* = 0.03) (43). These results argue against dUCBT in pediatric patients outside of clinical trials.

Further data continues to be reported on retrospective comparisons of CB versus other sources of hematopoietic stem cells for transplant. More recent studies incorporate high-resolution class I HLA typing for the controls. Brunstein and Collaborators compared the outcomes of patients receiving matched related donor transplants (*n* = 204), MUD (*n* = 152), mismatched unrelated donor (*n* = 52), and 4–6/6 HLA-matched dUCBT (*n* = 128). dUCBT was associated with similar LFS, at the expense of delayed hematopoietic engraftment, with lower aGVHD rates. Interestingly, relapse risk was lower in the dUCBT group, but at the expense of higher rates of early TRM (44).

Many of the above studies stress the importance of both HLA matching and TNC, however, the question arises: which is more important? In 2010, Avery analyzed 84 dUCBT and found no relation to HLA matching and sustained donor engraftment or time to neutrophil engraftment (45). In regards to single UCBT, both Arcese et al. and Laughlin et al. found no correlation with GVHD or survival with HLA disparity (17, 46). Thus it would seem that cord selection would be based primarily on TNC dose. However, a large retrospective analysis by Barker, focusing on 1061 single unit UCBT, found that patients with 0 HLA mismatches had a lower TRM independent of TNC. Interestingly, the greater HLA mismatch, the greater the importance of TNC was on overall survival. For example, patients with 1–2 HLA mismatch and a TNC >5.0 × 10^7^ c/kg had a TRM of almost half of patients with 1–2 HLA mismatch and a TNC between 2.5 and 4.9 × 10^7^ c/kg (47). This study suggests HLA mismatch plays a more important role that previously thought, especially in single UCBT. Current opinion with regard to cord selection recommends screening for grafts with >2.0 × 10^7^ c/kg and 4–6 HLA matches, then grouping cords into 6/6, 5/6, and 4/6 HLA-matched groups, and finally out of that best matched group, select the graft with the highest TNC (48).

## Umbilical Cord Enhancement

Focus has since shifted to methods of umbilical cord graft enhancement. These endeavors include umbilical cord expansion to increase the transplanted cell dose; methods that increase engraftment; alternative UCB delivery procedures; or co-transplantation with other types of cell grafts. As mentioned previously, due to a much larger body mass, the ratio of CD34+ cells or TNC to kilograms is significantly decreased in adults compared to pediatric patients. UCB expansion techniques attempt to overcome this challenge by increasing cell dose prior to infusion. Alternative delivery methods of UCB attempt to by-pass presumed stem cell trapping and/or decrease transit time to the bone marrow. Most of the studies reported are phase I or II and represent the latest advances in UCBT.

### Combining haploidentical hematopoietic stem cells with unrelated cord blood

Fernández and Collaborators hypothesized that selected CD34 haploidentical cells, when co-infused with UCB cells, would result in earlier engraftment. Interestingly, in the majority of the cases the haploidentical cells provided early engraftment but were subsequently rejected by the long-term engrafting cord blood cells (49). Recently, Lui and Colleagues expanded on Fernández’s observation (50). Forty-five patients with hematologic malignancies underwent RIC (fludarabine, melphalan, and ATG), followed by T cell depleted haploidentical CD34+ cells from a non-maternal donor (CD34+ cell dosage up to 3 × 10^6^ c/kg), and an UCB graft (minimum cell dose of 1 × 10^7^ c/kg and at least a 4/6 HLA match). Median time to neutrophil and platelet engraftment was 11 and 19 days, respectively, while aGVHD rate was 25%. TRM at day 100 was 9%, and 1-year overall survival was 55%.

### *Ex vivo* umbilical cord expansion

Shpall et al. employed a liquid culture system to expand a fraction of the UCB unit. Expanded UCB cells were selected for CD34, and after 10 days in liquid culture, TNC was expanded 56-fold, and CD34+ cells, fourfold. The expanded portion was then infused with the unmanipulated UCB. The median CD34+ cell dose was 10.4 × 10^4^ c/kg. The median time to neutrophil and platelet engraftment was 28 and 106 days respectively. Overall survival was 35% at 30 months (51). Subsequently, a randomized study at MDACC compared “standard” dUCBT versus one unmanipulated unit co-infused with a 100% expanded unit using the approach described above. Modifications of the expansion strategy included CD133 selection instead of CD34 (given the possibility of selecting earlier hematopoietic progenitors using CD133), and freezing the unselected cells left after CD133 separation, with reinfusion at the time of transplant (hypothesizing that this cell population could contain engraftment-facilitating cells). This trial, reported in abstract form, did not show an advantage for *ex vivo* expansion in terms of engraftment or survival (52). It is likely that cytokine-based liquid culture systems, such as the ones used in these expansion methods, push progenitor cells toward a less differentiated stage of maturation, essentially depleting the stem cell potential.

### *Ex vivo* umbilical cord expansion co-cultured with mesenchymal stem cells

The bone marrow microenvironment is important for the proliferation and differentiation of hematopoietic stem cells (53–58). Mesenchymal stem cells (MSCs) are found in the bone marrow and give rise to bone marrow stroma as well as other mesodermal tissues (59–62). Adding a layer of third party MSCs to the liquid culture system, as proposed by McNiece, Robinson, and Shpall, increased significantly the CD34 cell expansion *in vitro* (53). In addition, by adding MSCs the authors were able to expand cells without CD34 cell selection, a step associated with significant cell loss in the previous studies summarized above. Outcomes of the resulting clinical trial have been reported (54). The approach was a dUCBT in which one of the cords was *ex vivo* expanded with MSC, while the other unit was transplanted unmanipulated. Thirty-one high-risk hematologic malignancy patients were included. Expansion increased TNC numbers by a median factor of 12.2 and CD34+ cells by a median factor of 30.1, leading to grafts with median cell dose of 5.8 × 10^7^ TNC/kg. Importantly, the median number of megakaryocyte and platelet progenitors was also increased in the expanded cord. The median time to neutrophil and platelet engraftment was 15 and 42 days, respectively. Chimerism studies indicated that the expanded unit provided early engraftment while the unmanipulated unit provided most of long-term engraftment, although a minority of patients had evidence of long-term expanded-unit hemopoiesis (although the unmanipulated unit predominated). Some of the challenges in this study included the realization that family derived MSC pose significant logistical and coordination problems, which were alleviated by using an “off the shelf” MSC source. An important question unanswered by this study is whether faster engraftment will lead to less transfusions and shorter hospital stay. This question and others of cost effectiveness will be addressed in an upcoming randomized trial. These results provide the rationale for an ongoing international randomized multicenter study comparing unmanipulated dUCBT versus dUCBT containing one *ex vivo* expanded unit as described above.

### Co-infusion of MSC with umbilical cord stem cells

To study the effect of MSC and UCB co-infusion *in vivo*, MacMillan conducted a promising phase I–II study in which parental expanded MSCs were co-infused with UCB in pediatric patients with high-risk leukemia following myeloablative conditioning (63). Among patients receiving MSC/UCB co-infusions, median neutrophil and platelet engraftment occurred 19 and 53 days after transplant, respectively. Grade II–IV aGVHD rate was 38%, while there was no cGVHD observed and OS was 75% at 1 year.

### Copper chelation

Cellular copper concentration modulates differentiation of cord blood hematopoietic stem cells (64, 65). Copper chelation has been used to arrest maturation during cord blood expansion, and may lead to expansion of primitive progenitors without differentiation to a less pluripotent cell (66, 67). The feasibility of *ex vivo* expansion using a copper chelator in a liquid *ex vivo* expansion system was investigated in a phase I/II study that included 10 advanced hematologic malignancy patients. UCB units frozen in two fractions had the smaller fraction cultured for 21 days in media with cytokines. The median CD34+ cell increase was sixfold, resulting in a mean time to neutrophil and platelet engraftment of 30 and 48 days, respectively. Primary graft failure occurred in one patient. Grade II–IV aGVHD occurred in 44% of cases, while survival after 100 days was 90% (68). An ongoing multicenter randomized trial is comparing single unit UCBT with or without partial *ex vivo* expansion through copper chelation.

### Notch-mediated expansion

Delaney and Collaborators have investigated a novel strategy of *ex vivo* expansion based on the knowledge that Notch signaling is involved in hematopoietic stem cell renewal and maintenance (69–73). Mouse transplant models using *ex vivo* Notch-mediated expansion of selected human umbilical cord CD34+ stem cells have resulted in improved hematopoietic engraftment (74). *Ex vivo* expansion of CD34+ HSC resulted in a 222-fold expansion following culture for 17–21 days in the presence of fibronectin fragments and immobilized engineered Notch ligand. A phase I clinical trial was conducted in Seattle, in which 10 hematologic malignancy patients with receiving myeloablative conditioning and dUCBT with HLA matched to at least 4/6 HLA loci in each unit. One unit was 100% *ex vivo* expanded and the other was not. The median time to neutrophil engraftment was 16 days; grade II–IV aGVHD was 100%. After a mean follow-up of 354 days, survival was 70%.

Since most studies reported so far used a dUCBT or investigated expansion of a fraction of a single unit (Table 4), it is unclear if expansion preserves the long-term engraftment capacity. The expanded unit is outcompeted in dUCBT or fractional models, as discussed above, but it is yet unknown whether this phenomenon represents loss of engraftment-facilitating cells (most expansion platforms deplete lymphocytes) or stem cell exhaustion.

**Table 4:** **Listing of different umbilical cord blood enhancement studies and outcomes**.

Study	Type	No. Patients	Conditioning regimen	GVHD prophylaxis	Cord information	Neutrophil (ANC = 500) and platelet engraftment (20,000)	Grade II–IV aGVHD (%)	Survival
Shpall et al. (51)	Cytokine expansion	37	High-dose chemotherapy	Cyclosporine + prednisone	Divided cord, at least HLA 4/6 matched	Neutrophil = day 28; platelet = day 106	40	13 month OS = 35%
De Lima et al. (68)	Copper chelation	10	Varied: melphalan + Fludarabine + rATG, fludarabine + busulfan + rATG	Tacrolimus + “mini” methotrexate	Divided cord, at least HLA 4/6 matched	Neutrophil = day 30; platelet = day 48	44	Day 100 OS = 90%
MacMillan et al. (40)	Parental MSC co-infusion, pediatric	15	Cyclophosphamide + 1350 cGy + hATG, for age < 1 year: mel/bu/hATG	CSA + methylprednisolone	One cord, at least 4/6 HLA matched	Neutrophil = day 19; platelet = day 53	38	1 year OS = 75%
Delaney et al. (74)	Notch-mediated UBC expansion	10	1320 cGy TBI, 120 mg/kg Cytoxan, 75 mg/m^2^ fludarabine		dUCBT, 1 cord notch expanded, at least 3/6 HLA matched	Neutrophil = day 16	100	Day 354 OS = 70%
De Lima et al. (54)	MSC expansion, dUCBT	31	Melphalan, thiotepa, fludarabine, rATG	Tacrolimus + MMF	One cord MSC expanded co-infused with other cord	Neutrophil = day 15; platelet = day 42	42	1 year OS = 32%

MSC, mesenchymal stem cell; dUCBT, double umbilical cord blood transplant; CSA, cyclosporine; hATG, horse anti-thymocyte globulin; rATG, rabbit anti-thymocyte globulin; MMF, mycophenolate mofetil; OS, overall survival.

### Intraosseous transplantation

A significant proportion of hematopoietic stem cells do not reach the bone marrow niche after intravenous (iv) infusion (75, 76). In mouse models, intrabone (ib) injection of hematopoietic stem cells improves engraftment, possibly by bypassing “trapping” in organs such as the lung, liver, and kidney (77–82). Interestingly, ib transplantation has also led to decreased GVHD rates in murine models (83–85).

Hägglund and Colleagues conducted the first human studies of ib transplantation (86). Thirty-four patients were randomized to either iv alone, ib alone, or iv + ib following myeloablative conditioning. There were no differences in survival, engraftment, or GVHD between groups. Of note, Technetium (Tc-99m) scintigraphy imaging studies demonstrated similar stem cell body distribution in all groups.

Frassoni and Collaborators performed 32 ibUCBT after myeloablative conditioning to treat hematologic malignancies (87). The grafts were matched to at least 4/6 HLA loci and the median TNC dose infused was 2.6 × 10^7^ c/kg. Median time to neutrophil and platelet engraftment was 23 and 27 days, respectively. There were no instances of grade III–IV aGVHD, and the authors suggested there was a reduced risk of aGVHD with this strategy. Interestingly, a subsequent study performed PET imaging sites of ibUCBT recipients and found that platelet recovery correlated with increased FDG uptake within the area of injection (88).

Studies performed by investigators at the University of Minnesota group were not able to reproduce these promising results. Brunstein et al. published a study of 10 patients who underwent dUCBT following myeloablative conditioning to treat hematologic malignancies (89). One unit underwent ib infusion, whereas the other was injected iv. The trial was closed earlier due to futility given engraftment times similar to historic numbers.

Recently, Carrancio et al. showed that ib co-transplantation of MSC in combination with human CD34+ UCB cells led to improved engraftment in a NOC/SCID mouse model (90). We are now also investigating these intriguing observations, with the hypothesis that human ib CB transplants can be improved by “priming” with MSC.

Following the preliminary success of many of the above studies, many new trials are enrolling patients. Most new trials have focused on novel methods of UCB engraftment or expansion.

## Ongoing and New Studies

Following the preliminary success of many of the above studies, many new trials are enrolling patients. Most new trials have focused on novel methods of UCB engraftment or expansion.

Gamida Cell is sponsoring multicenter studies investigating *ex vivo* expansion utilizing NiCord (copper chelation). One trial is a dUCBT following myeloablative conditioning: one cord will be 100% expanded while the other will be unmanipulated. They are also sponsoring a trial evaluating the safety of a single UCBT in which a fraction of the unit is expanded with NiCord (Clinical Trial identifier: NCT01590628, NCT01221857).

The University of Pennsylvania has opened a phase I study where patients undergoing UCBT will have a part of the cord expanded specifically for the generation of T cells that could be used for adoptive immunotherapy after transplant (Clinical Trial identifier: NCT00891592).

The Fred Hutchinson Cancer Center has opened a randomized phase II trial comparing dUCBT with or without Notch-mediated *ex vivo* cord expansion (Clinical Trial identifier: NCT01690520). Another phase II study opened at that institution is investigating the infusion of a non-HLA-matched *ex vivo* expanded cord (i.e., an “off the shelf” cord) combined with either a single or dUCBT (Clinical Trial identifier: NCT01175785). Both studies focus on reducing engraftment time.

Dana Farber Cancer Institute investigators are exposing UCB grafts to PGE2 with the goal of improving engraftment rates (Clinical Trial identifier: NCT00890500).

The MD Anderson Cancer Center group (P.I. Elizabeth Shpall) and Mesoblast are leading a randomized trial of dUCBT in which one unit is *ex vivo* expanded using the MSC strategy discussed above, versus controls receiving unmanipulated dUCBT grafts. This trial is also open for accrual at University Hospitals Case Medical Center, Case Western Reserve University, and other centers in the U.S. and Europe (Clinical Trial Identifier: NCT00498316).

Shpall and Collaborators are studying fucosylation as a means of increasing homing and engraftment of UCB. Fucosylation is the process of adding fructose to a molecule. *Ex vivo* fucosylation of cord blood CD34+ cells has shown to improve engraftment in a NOD/SCID mouse model (91). In this trial, the UCB unit is incubated with a fucosylating enzyme before transplantation. It hypothesized that fucosylation may lead to faster engraftment in humans by changing UCB CD34+ cell ligands (Clinical Trial Identifier: NCT01471067).

## Conclusion

Over the last 25 years, UCBT has become a valid source of hematopoietic stem cells, allowing for increased access to hematopoietic stem cell transplantation, particularly in patients without available BMT or PBSC donors. Decreased engraftment rates and kinetics are still important limitations of this hematopoietic stem cell source. UCB engraftment has been the focus of intensive basic and early clinical research and recent progress indicates that several methods will become soon widely available to increase the applicability of this graft type. Future challenges to research in UCBT will include identification of the optimal methods that expedite engraftment and immune reconstitution after transplant and the effect these methods will have in GVHD, disease control, and long-term transplant complications. With the increased development of haploidentical donor transplants, researchers will have the additional charge of performing studies that accurately delineate the role of these two alternative graft sources to allow a rational use of resources in the new exciting reality of widespread availability of graft sources for patients in need of an allogeneic hematopoietic stem cell transplantation.

## Conflict of Interest Statement

The authors declare that the research was conducted in the absence of any commercial or financial relationships that could be construed as a potential conflict of interest.
